# UNTREATED OBSTRUCTIVE SLEEP APNEA INCREASES HOSPITALIZATION RISK IN MEDICARE BENEFICIARIES 65+ WITH HEART DISEASE

**DOI:** 10.1093/geroni/igac059.2532

**Published:** 2022-12-20

**Authors:** Jennifer Kirk, Emerson Wickwire, Virend Somers, Jennifer Albrecht

**Affiliations:** University of Maryland, Baltimore, Annapolis, Maryland, United States; University of Maryland, School of Medicine, Balrtimore, Maryland, United States; Mayo Clinic, Rochester, Minnesota, United States; University of Maryland, School of Medicine, Baltimore, Maryland, United States

## Abstract

Cardiovascular disease (CVD) is a leading cause of death and disability in the United States and worldwide. Untreated obstructive sleep apnea (OSA) is common among older adults with CVD and may increase their risk of hospitalization. However, prior research has been limited by smaller samples of participants undergoing diagnostic sleep testing. Our objective was to estimate the effect of untreated OSA on the risk of hospitalization among older Medicare beneficiaries with CVD age 65+. Using a 5% sample of Medicare administrative claims data from 2006 to 2013, beneficiaries were identified as cases diagnosed with OSA or controls without any sleep-related diagnoses. Then, we restricted only to beneficiaries with a CVD diagnosis by the index date (i.e., OSA diagnosis date or matched date in controls). Next, we assessed the risk of hospitalization, counting only one per beneficiary, during the year before the index date. Because OSA is typically present long before it is diagnosed, the year before OSA diagnosis was assumed to represent a period of untreated OSA. Covariates were balanced between cases and controls using inverse probability of treatment weighting (IPTW). Then using logistic regression, we estimated the risk of hospitalizations the year prior to the index date. Of the 142,893 beneficiaries with CVD, only 19,390 were diagnosed with OSA. In the IPTW model, beneficiaries with untreated OSA were more likely to be hospitalized (odds ratio, 1.82; 95% CI 1.77–1.87). Results suggest screening for OSA among Medicare beneficiaries with CVD may identify older adults at increased risk of hospitalization.

